# Working Memory Reloaded: Tyrosine Repletes Updating in the *N*-Back Task

**DOI:** 10.3389/fnbeh.2013.00200

**Published:** 2013-12-16

**Authors:** Lorenza S. Colzato, Bryant J. Jongkees, Roberta Sellaro, Bernhard Hommel

**Affiliations:** ^1^Institute for Psychological Research, Leiden Institute for Brain and Cognition, Leiden University, Leiden, Netherlands

**Keywords:** working memory, tyrosine, dopamine, updating

## Abstract

In this study we tested the idea that the food supplement l-Tyrosine (TYR) repletes resources required for cognitive-control operations. We investigated whether the “updating” (and monitoring) of working memory (WM) representations, a key cognitive-control function, can be promoted by administering TYR, the biochemical precursor of dopamine. Participants performed an *N*-back task where we compared the WM-demanding 2-back condition with the WM-undemanding 1-back condition. As expected, TYR promoted performance in the more demanding (2-back) but not in the easier (1-back) condition, suggesting that TYR selectively targets cognitive-control operations. This result suggests that TYR can replete cognitive resources when more control is needed and, more generally, that food can act as a cognitive enhancer.

## Introduction

In a seminal essay (1862/1960), the German philosopher Ludwig Feuerbach claimed that “Der Mensch ist, was er ißt” (you are what you eat). Feuerbach was probably the first intellectual to promote the idea that the food one eats has a bearing on one’s state of mind. This idea later became the motto of the hippie culture, which promoted eating organic and healthy food. Since then, the idea that the food we eat modulates the way we think and perceive the world has been very suggestive in popular culture and the focus of scientific research. One of the most investigated amino acids (building blocks of proteins) is tyrosine (TYR), which is contained in food such as fish, soy, eggs, milk, and bananas. In healthy population the average daily intake of TYR is 7 mg/kg, one half of the (World Health Organization) WHOs upper requirement of 14 mg/kg (see Deijen, 2005). Importantly, tyrosine is the biochemical precursor of two important brain catecholamine neurotransmitters: norepinephrine (NE) and dopamine (DA). The supplementation of TYR, or TYR-containing diets, increase plasma TYR and enhance brain DA and NE release (Lehnert et al., 1984; Reinstein et al., 1984; Acworth et al., 1988; During et al., 1988; see Deijen, 2005, for a comprehensive review). Glaeser et al. (1979) found, after the administration of a single oral dose of 100 mg/kg TYR, that mean plasma TYR levels were maximal after 2 h, rising from 69 ± 3.9 to 154 ± 9.5 nmol/ml. Previous literature has mainly focused on the role of TYR as “counteractor” of conditions that cause brain DA and NE depletion, such as stress (Deijen and Orlebeke, 1994; Shurtleff et al., 1994; Mahoney et al., 2007). Only in one study TYR has been administered without exposure to stress (Thomas et al., 1999), using a multiple task battery (SYNWORK1; Elsmore, 1994) designed to measure working memory (WM), arithmetic skills, and visual and auditory monitoring simultaneously, and a simple task battery consisting of only two of the subtasks: the Sternberg Memory task (Sternberg, 1966) and the Visual Monitoring task (Loeb and Binford, 1968). The results revealed beneficial effects of TYR supplementation when competing requirements to perform other tasks simultaneously degrade performance (Thomas et al., 1999). This indicates that TYR may replete cognitive resources, but only under sufficiently demanding conditions.

Interestingly, executive control has been considered to emerge from the interplay between cognitive stability (defined as the maintenance of task-relevant representations) and flexibility (defined as the ability to adapt, update, and shift between informational states) – two major, but partially antagonistic functions of cognitive control, related correspondingly to the prefrontal cortex (PFC) and the striatum, which both are modulated by DA (Cools, 2006; Cools and D’Esposito, 2010) – the precursor of which is TYR. According to Cools (2006), the same high DA levels in the PFC that are beneficial for the stability of representations may reduce the ability to flexibly alter cognitive representations. Low DA levels in the PFC may in turn be beneficial for the flexible updating of cognitive representations, but at the same time impair the ability to maintain representations in the face of intervening distractors. Several studies have shown that striatal dopamine plays a crucial role in WM updating. According to Moustafa et al. (2008), the nigrostriatal dopaminergic pathway serves as a gate to signal when and when not to update information in prefrontal WM. Consistent with this idea, Siessmeier et al. (2006) found that administering DA agents to healthy subjects led to a correlation between DA uptake in the striatum and BOLD activity in the dorsolateral PFC, suggesting that the striatum might drive WM activity in the PFC. Moreover, a PET study showed that individual WM capacity predicts the striatal dopamine synthesis capacity: subjects with low WM capacity have a low synthesis capacity while subjects with high WM capacity have a high synthesis capacity (Cools et al., 2008).

The current study focused, for the first time, on the acute effect of TYR supplementation on the updating (and monitoring) of WM representations – a key cognitive-control function (Miyake et al., 2000). We tested whether WM updating can be promoted by administering the food supplement TYR. We investigated the link between TYR supplementation and the monitoring of WM in healthy adults exposed to an oral dose of either TYR or a neutral placebo. WM updating was measured by the *N*-back task: participants were required to decide whether each stimulus in a sequence matched the one that appeared *n* items ago. In conditions with *n* = 2 or higher (where the match is for the stimulus that appeared two or more items ago), this task requires the on-line monitoring and updating of WM content, which is known to be cognitively demanding (Kane et al., 2007). In contrast, performance in the easiest 1-back condition (where the match is for the previous item) can rely on immediate perceptual priming (as the two matching items appear in direct succession), which makes this condition a suitable (i.e., WM-undemanding) control condition. Accordingly, we assumed that the depletion of cognitive resources would affect performance in the *N*-2 condition more than performance in the less demanding *N*-1 condition. If so, and if the hypothesized repleting effect of TYR is really restricted to cognitively challenging conditions (Anisman and Sklar, 1979), the positive impact of TYR should be stronger in the more demanding 2-back condition than in the easier 1-back condition.

## Materials and Methods

### Participants

Twenty-two undergraduate students of the Leiden University (all females, mean age = 19.7 years, range 18–25; mean Body Mass Index = 21.5, range 18–24; all right-handed) with no cardiac, hepatic, renal, neurological or psychiatric disorders, personal or family history of depression, migraine, and medication or drug use participated in the experiment. Participants were selected individually via a phone interview by the same lab-assistant using the Mini International Neuropsychiatric Interview (M.I.N.I.; Sheehan et al., 1998). The M.I.N.I. is a well-established brief diagnostic tool in clinical and stress research that screens for several psychiatric disorders and drug use (Sheehan et al., 1998; Elzinga et al., 2007). Psychoactive substance use was not assessed.

Written informed consent was obtained from all subjects; the protocol and the remuneration arrangements of 20 euro in cash payment were approved by the local ethical committee (Leiden University, Institute for Psychological Research).

In separate sessions participants were exposed to either an oral dose (powder) of 2.0 g of l-Tyrosine (TYR) (supplied by Bulkpowders Ltd.) or of 2.0 g of microcrystalline cellulose (Sigma-Aldrich Co. LLC), a neutral placebo, dissolved in 400 ml of orange juice. The two sessions were separated by 3–7 days. A double blind, placebo-controlled, randomized cross-over design with counterbalancing of the order of conditions was used to avoid expectancy effects.

Following Markus et al. (2008) women using contraception were tested when they actually used the contraception pill. On each experimental morning, participants arrived at the laboratory at 9:30 a.m. Participants had been instructed to fast overnight; only water or tea without sugar was permitted. In addition, subjects were not allowed to use any kind of drugs before and during the experiment or to drink alcohol the day before their participation and arrival at the laboratory. Thirty minutes after the administration of either TYR or the neutral placebo participants were allowed to eat an apple.

### Apparatus and procedure

All participants were tested individually. Upon arrival, participants were asked to rate their mood on a 9 × 9 Pleasure × Arousal grid (Russell et al., 1989) with values ranging from −4 to 4. Heart rate (HR) and systolic and diastolic blood pressure (SBP and DPB) were collected from the non-dominant arm with an OSZ 3 Automatic Digital Electronic Wrist Blood Pressure Monitor (Spiedel and Keller). One hour following the administration of TYR (corresponding to the beginning of the 1 h-peak of the plasma concentration; Glaeser et al., 1979) or placebo, participants rated again their mood before having HR, SBP, and DBP measured for the second time. Immediately after, participants were asked to perform the *N*-back task.

After completing the *N*-back task, participants again rated their mood before having HR, SBP, and DBP measured for the third time.

#### *N*-back task

The experiment was controlled by a ACPI uniprocessor PC running on an Intel Celeron 2.8 GHz processor, attached to a Philips 109B6 17″ monitor (LightFrame 3, 96 dpi with a refresh rate of 120 Hz). Responses were made by pressing the left shift-key and the right shift-key of the QWERTY computer keyboard with the left and right index finger, respectively. Stimulus presentation and data collection were controlled using E-Prime 2.0. Software system (Psychology Software Tools, Inc., Pittsburgh, PA, USA).

The two conditions of the *N*-back task were adopted from Colzato et al. (2009). A stream of single visual letters (taken from B, C, D, G, P, T, F, N, L) was presented (stimulus–onset asynchrony 2000 ms; duration of presentation 1000 ms). Participants responded to targets (presented in 33% of the trials) and to non-targets.

Half of the participants pressed the left shift-key in response to a target and the right shift-key in response to a non-target; the other half of the participants received the opposite mapping. Target definition differed with respect to the experimental condition. In the 1-back condition, targets were defined as stimuli within the sequence that were identical to the immediately preceding one. In the 2-back condition, participants had to respond if the presented letter matched the one that was presented two trials before. Each condition consisted of 18 practice trials followed by two blocks of 52 stimuli each. All participants performed the 1-back condition first and then the 2-back condition. Stimuli presentation was pseudo-randomized to avoid the occurrence of lure trials (i.e., non-target letters that match a recent letter in the sequence but not the letter *N*-back), which are known to elicit more false alarms (FA) and misses than non-lure non-targets because of their familiarity and resemblance to targets (see Kane et al., 2007).

### Statistical analyses

Mood, HR, BPS, and BPD were analyzed separately by means of repeated-measures analyses of variance (ANOVAs) with condition (TYR vs. Placebo) and effect of time (first vs. second vs. third measurement) as within-subjects factor. For the *N*-back task, repeated-measures ANOVAs with load (1-back vs. 2-back) and condition (TYR vs. Placebo) as within-subjects factors were carried out on reaction times (RTs) on correct trials as well as accuracy, hits, correct rejections, FA, and misses in percent. Furthermore, the sensitivity index *d*′ was calculated for both experimental conditions and the two WM loads separately (see Haatveit et al., 2010; Buckert et al., 2012). This index, which derives from signal detection theory (Swets et al., 1961), provides a combined measure of correct hits and FA and thus reflects participants’ ability to discriminate target from non-targets, with higher *d*′ indicating better signal detection. *d*′ was computed from hit rate and FA rate using the following formula: *Z*_HIT_ − *Z*_FA_, where *Z* represents the *z*-scores of the two rates (Macmillan and Creelman, 1991). The *Z* transformation was done using the inverse cumulative distribution function in Microsoft Excel 2007 (NORMSINV). Perfect scores were adjusted using these formulas: 1 − 1/(2*n*) for perfect (i.e., 100%) hits, and 1/(2*n*) for zero FA, where *n* was number of total hits or FA (Macmillan and Creelman, 1991).

Data analyses were performed using the Statistical Package for the Social Science (SPSS) for Windows, Version 21.0 (SPSS Inc., Chicago, IL, USA). A significance level of *p* < 0.05 was adopted for all statistical tests.

## Results

### *N*-back task

Table 1 shows mean RTs (in ms), hits, correct rejections, FA, and misses (in percent) for the *N*-back task separately for placebo and TYR conditions.

**Table 1:** **Mean RTs (in ms), hits, correct rejections, false alarms, and misses (in percent) for the *N*-back task for placebo and TYR conditions**.

*N*-back (WM monitoring/updating)	Placebo	TYR
**1-BACK**		
Reaction times (ms)	494 (14.9)	462 (11.3)
Hits (%)	90.5 (1.5)	92.3 (1.5)
Correct rejections (%)	95.6 (0.8)	95.8 (0.6)
False alarms (%)	4.4 (0.8)	4.2 (0.6)
Misses (%)	9.5 (1.5)	7.7 (1.5)
**2-BACK**		
Reaction times (ms)	567 (20.1)	520 (16.5)
Hits (%)	82.8 (2.8)	88.2 (1.7)
Correct rejections (%)*	89.6 (1.6)	94.3 (1.0)
False alarms (%)*	10.9 (1.5)	6.7 (1.1)
Misses (%)	17.2 (2.8)	11.8 (1.7)

Significant difference between the two conditions; *p < 0.05. SE are shown within parentheses.

Load affected all dependent measures, showing that higher load increased RTs, *F*(1, 21) = 61.09, *p* = 0.0001, MSE = 1547.315, $\eta_{p}^{2}=0.74,$ and reduced accuracy, *F*(1, 21) = 14.00, *p* = 0.001, MSE = 30.774, $\eta_{p}^{2}=0.40.$ Higher load also produced fewer hits, *F*(1, 21) = 8.94, *p* = 0.007, MSE = 85.638, $\eta_{p}^{2}=0.30,$ and correct rejections, *F*(1, 21) = 19.84, *p* = 0.0001, MSE = 15.592, $\eta_{p}^{2}=0.49,$ but more FA, *F*(1, 21) = 29.30, *p* = 0.0001, MSE = 15.255, $\eta_{p}^{2}=0.58,$ and misses, *F*(1, 21) = 8.94, *p* = 0.007, MSE = 85.638, $\eta_{p}^{2}=0.30,$ than the lower load did. With regard to the effect of condition, the intake of TYR, as compared to placebo, reduced significantly RTs, *F*(1, 21) = 6.67, *p* = 0.02, MSE = 5188.740, $\eta_{p}^{2}=0.24,$ produced fewer misses, *F*(1, 21) = 5.27, *p* = 0.03, MSE = 54.864, $\eta_{p}^{2}=0.20,$ and higher hits, *F*(1, 21) = 5.27, *p* = 0.03, MSE = 54.864, $\eta_{p}^{2}=0.20,$ but it did not affect the number of FA, correct rejections, and accuracy (*F*’s ≤ 2.54, *p*’s ≥ 0.07).

**Figure 1:**
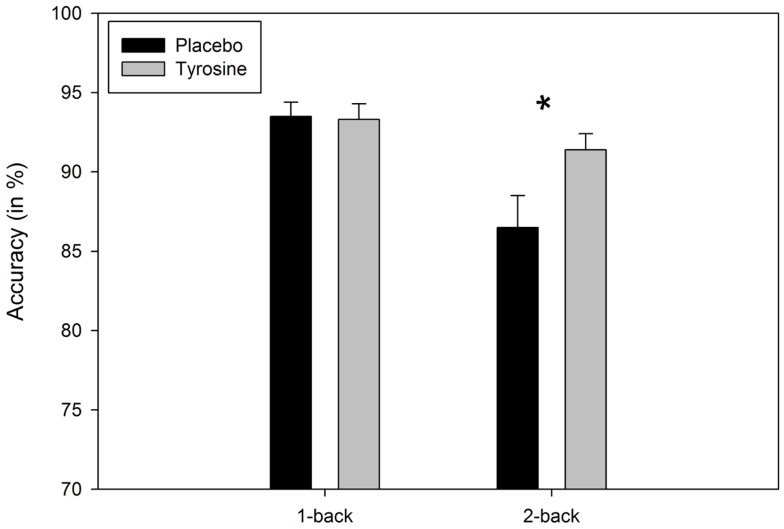
**Mean Accuracy (in %) as a function of load (1-back vs. 2-back) and condition (Placebo vs. TYR)**. Asterisk indicates significant (*p < 0.05) effect of TYR on the 2-back task. Vertical capped lines atop bars indicate SE of the mean.

Most importantly, significant interactions between load and condition were observed for FA, *F*(1, 21) = 6.43, *p* = 0.02, MSE = 13.800, $\eta_{p}^{2}=0.23,$ correct rejections, *F*(1, 21) = 7.31, *p* = 0.01, MSE = 15.039, and, as expected, for accuracy, *F*(1, 21) = 11.04, *p* = 0.003, MSE = 12.654, $\eta_{p}^{2}=0.35.$ In the 1-back condition, participants’ performance after placebo and TYR was comparable (4.4 vs. 4.2% FA, 95.6 vs. 95.8% correct rejections, and 93.5 vs. 93.3% correct responses), *t*’s < 1, $\left[{d\prime }_{\mathrm{(placebo)}}=33,\;{d\prime }_{\mathrm{(TYR)}}=3.5\right].$ In contrast, in the 2-back, the intake of TYR reduced significantly the percentage of FA (10.9 vs. 6.7%, after placebo and TYR, respectively), *t*(21) = 2.29, *p* = 0.03, and increased significantly the number of correct rejections (89.6 vs. 94.3%, after placebo and TYR, respectively), *t*(21) = 2.47, *p* = 0.02, and participants’ accuracy (86.5 vs. 91.4% after placebo and TYR, respectively), *t*(21) = 2.54, *p* = 0.02, see Figure 1, $\left[{d\prime }_{\mathrm{(placebo)}}=2.4,\;{d\prime }_{\mathrm{(TYR)}}=2.9\right].$

### Physiological and mood measurements

ANOVAs revealed that HR (73 vs. 67 vs. 65 and 71 vs. 68 vs. 63 after placebo and TYR, respectively), BPD (70 vs. 66 vs. 68 and 68 vs. 71 vs. 68 after placebo and TYR), BPS (112 vs. 111 vs. 110 and 113 vs. 113 vs. 109 after placebo and TYR), and mood (1.4 vs. 1.6 vs. 1.2 and 1.7 vs. 1.7 vs. 1.5 after placebo and TYR) did not significantly change after the intake of TYR, *F*’s < 1. This suggests that we can rule out an account of our results in terms of physiological and mood changes.

## Discussion

The present study is the first to demonstrate that TYR supplementation promotes WM updating. As expected, the more challenging 2-back condition was more sensitive to the effect of TYR, which reinforces our suspicion that only tasks with considerable cognitive demands benefit from TYR. As we have argued in the introduction, this may be because more demanding cognitive operations are more likely or more efficient to exhaust the available cognitive resources, which can then be repleted by TYR. The idea that cognitive-control operations are particularly likely to exhaust cognitive resources fits with the concept of “ego-depletion” suggested by Baumeister et al. (1998), which would suggest that TYR can be used as an effective “ego-repletor.”

Our results support the materialist approach that “you are what you eat” (Feuerbach, 1862/1960). The food we eat may thus act as a cognitive enhancer that modulates the way we deal with the physical world, but at least with our own short-term memory traces. At short-term, the supplementation of TYR or consuming TYR-rich food to improve cognitive processes is safe and healthy and can be regarded as an alternative to cognitive-enhancing drugs, such as Ritalin or Modafinil, the use of which is quite popular among students to improve their academic performance. In contrast to TYR, these prescribed drugs do not have FDA (U.S. Food and Drug Administration) GRAS (Generally Recognized as Safe) status and are often associated with side effects commonly experienced with amphetamine-like drugs.

In view of the evidence that striatal dopaminergic pathways play a major role in the updating of WM (Siessmeier et al., 2006; Cools et al., 2008; Moustafa et al., 2008), our findings further suggest that TYR is involved in the monitoring of cognitive representations. A limitation of our study is the lacking of plasma TYR levels measurements. In a replication of our study, it would be important to correlate those assessments with the accuracy performance in the 2-back condition.

Future research needs to explore the direct effect of prolonged use of tyrosine supplementation on the brain. It remains to be demonstrated, for instance, that tyrosine use produces long-term changes at the neuromodulatory (enhanced functioning of DA receptors) and at functional level (in PFC and striatum) proportionally to the degree of behavioral performance enhancements. Moreover, it will be also important to take individual differences into account. There is ample evidence suggesting a considerable role for individual differences with respect to the efficiency of cognitive-control processes and the neurotransmitter systems driving them (Cools, 2006). Furthermore, in healthy humans tyrosine has been shown to reverse stress-induced deficits in WM and attentional tasks, but in particular in individuals who were most affected by the stressors (Deijen and Orlebeke, 1994; Shurtleff et al., 1994; Mahoney et al., 2007) – suggesting individual differences in the reactivity to tyrosine. It makes sense to assume that preexisting neuro-developmental factors (such as genetic variability related to levels of the neurotransmitter systems) affect the degree to which individuals can benefit from tyrosine supplementation, especially because many of them are arguably tapping into cognitive-control processes.

In sum, even if long-term effects are yet to be demonstrated, our findings suggest that the supplementation of TYR, or TYR-containing diets, may promote cognitive enhancement in inexpensive, efficient, and healthy ways.

## Conflict of Interest Statement

The authors declare that the research was conducted in the absence of any commercial or financial relationships that could be construed as a potential conflict of interest.
